# Cryptochrome 1 Overexpression Correlates with Tumor Progression and Poor Prognosis in Patients with Colorectal Cancer

**DOI:** 10.1371/journal.pone.0061679

**Published:** 2013-04-23

**Authors:** Hongyan Yu, Xiangqi Meng, Jiangxue Wu, Changchuan Pan, Xiaofang Ying, Yi Zhou, Ranyi Liu, Wenlin Huang

**Affiliations:** 1 State Key Laboratory of Oncology in South China, Cancer Center, Sun Yat-sen University, Guangzhou, People's Republic of China; 2 CAS Key Laboratory of Pathogenic Microbiology and Immunology, Institute of Microbiology, Chinese Academy of Science, Beijing, People's Republic of China; 3 Medical Oncology, Sichuan Cancer Hospital and Institute, Second People′s Hospital of Sichuan Province, Cheng Du, People's Republic of China; The University of Hong Kong, China

## Abstract

**Background:**

Clock genes drive about 5–15% of genome-wide mRNA expression, and disruption of the circadian clock may deregulate the cell's normal biological functions. Cryptochrome 1 is a key regulator of the circadian feedback loop and plays an important role in organisms. The present study was conducted to investigate the expression of Cry1 and its prognostic significance in colorectal cancer (CRC). In addition, the function of Cry1 in human CRC was investigated in cell culture models.

**Methods:**

Real-time quantitative PCR, Western blot analysis and immunohistochemistry were used to explore Cry1 expression in CRC cell lines and primary CRC clinical specimens. MTT and colony formation assays were used to determine effects on cellular proliferation ability. The animal model was used to explore the Cry1 impact on the tumor cellular proliferation ability *in vivo*. Transwell assays were performed to detect the migration ability of the cell lines. Statistical analyzes were applied to evaluate the diagnostic value and the associations of Cry1 expression with clinical parameters.

**Results:**

Cry1 expression was up regulated in the majority of the CRC cell lines and 168 primary CRC clinical specimens at the protein level. Clinical pathological analysis showed that Cry1 expression was significantly correlated with lymph node metastasis (*p* = 0.004) and the TNM stage (*p* = 0.003). High Cry1 expression was associated with poor overall survival in CRC patients (*p* = 0.010). Experimentally, we found that up-regulation of Cry1 promoted the proliferation and migration of HCT116 cells, while down-regulation of Cry1 inhibited the colony formation and migration of SW480 cells.

**Conclusions:**

These results suggest that Cry1 likely plays important roles in CRC development and progression andCry1 may be a prognostic biomarker and a promising therapeutic target for CRC.

## Introduction

Circadian rhythms are daily oscillations in various biologic processes. In recent years, regulation of circadian rhythm has become better understood. The molecular mechanism of circadian oscillations in the suprachiasmatic nuclei (SCN) and peripheral cells is based on the feedback loops of eight core circadian genes [1], [2]: *Period1 (Per1)*, *Period2 (Per2)*, *Period3 (Per3)*, *Clock*, *Bmal1*, *Casein Kinase I ε (CKIε)*, *Cryptochrome1 (Cry1) and Cryptochrome2 (Cry2)*. Among the eight genes, the Crys accumulate in the cytoplasm and then enter the nucleus, promoting the formation of stable Per/Cry/CK1ε complexes. Once in the nucleus, the Crys break apart the Bmal1/Clock-associated transcriptional complex, resulting in inhibition of Cry and Per transcription and derepression of Bmal1 transcription. The molecular clock of the peripheral tissues coordinates the transcription of the circadian genes. The circadian genes are largely tissue specific and link key tissue functions to the circadian environment, allowing these important functions to be available at specific times when they are most needed[3], [4], [5].

In mammals, about 5–15% of genome-wide mRNA expression is driven by circadian genes, including key cell cycle regulators (such as *cyclin D1*), oncogenes, and tumor suppressors, such as *c-myc*and*WEE-1* (a kind kinase that blocks cell division) [6]. Disruption of the circadian clock may deregulate normal cellular biological functions and have significant effects on human health, causing conditions such as sleep disorders, gastrointestinal and cardiovascular illnesses, and depression. The circadian clock is also associated with an increased incidence of several epithelial cancers [7], [8], [9], [10], [11], [12]. In mouse models, transplanted tumors grow twice as fast in SCN-lesioned mice as in sham-lesioned animals [13]. These findings suggest that a close connection exists between circadian organization and the development of various cancers. Relations between circadian genes and cancer have been demonstrated in recent years. The host circadian clock has been reported to play an important role in the endogenous control of tumor progression [14].Cry1, one member of the Cryptochrome family, has been shown to be essential to the negative arm of the circadian feedback loop [15], [16].

Colorectal cancer (CRC) is one of the three leading causes of cancer-related mortality worldwide [17]. CRC patients frequently develop lymph node metastases during the early stages of the disease. During the advanced stages, the majority of patients also develop liver, lung and peritoneum metastases. The factors involved in CRC metastasis are largely unknown; however, the dysregulation of molecular processes is considered to result in the growth and metastasis of CRC. Consequently, the importance of markers that promote the development of CRC has been emphasized, as they might provide therapeutic targets [18], [19].

However, studies assessing the relations of Cry1 expression to clinicopathological features and outcomes in colorectal cancer have not been reported. We, therefore, evaluated the expression levels of Cry1 in human colorectal cancer tissues and matched non-tumor mucosa and analyzed the clinical significance of Cry1 expression inCRC patients. Our data demonstrate that Cry1 expression is significantly correlated with the TNM stage and lymph node status. Thus, Cry1 may serve as a new diagnosis marker and therapeutic target for CRC therapy.

## Materials and Methods

### Patients and follow-up

One hundred and sixty-eight colorectal cancer patients of all TNM stages at the Cancer Center of Sun Yat-sun University between September 1999 and December 2005 were included in the study. Ten paired tissues from the 168 samples were used for a q-PCR assay.

The study was approved by the Ethics Committee of the Sun Yat-sen University Cancer Center Institutional Board, and written informed consent was obtained from all of the patients.

Clinical data, including age, gender, tumor size, tumor location,preoperative carcinoembryonic antigen (CEA) levels and carbohydrate antigen 19-9 (CA199) levels, were collected from unprocessed case reports.

Pathological parameters, such as tumor invasive depth, differentiation grade and histological pattern were collected from pathological reports and checked by pathologists. The patients were followed-up once every three months for the first two years, once every six months during the third and fourth years, and once a year after the fifth year postoperatively.

All patients were contacted by phone to check on their health status; the last follow-up date was March 1, 2012. The disease-free survival (DFS) and overall survival (OS) times were computed from the operation date to metastasis or recurrence date or the date of death, or the last censor time.

### Cell culture

The human CRC cell lines SW480, SW620, HCT116, HT29, the human embryonic kidney cell line GP293 and the normal colon epithelium cell line FHC were obtained from the American Type Culture Collection. The THC8307 cells were obtained from our own laboratory collection. The CRC cell lines were grown in DMEM supplemented with 10% fetal bovine serum (FBS; Invitrogen, Carlsbad, CA). The FHC cells were grown in DMEM: F12 medium containing 0.005 mg/ml insulin, 10 ng/ml cholera toxin, 100 ng/ml hydrocortisone and 0.005 mg/ml transferrin and supplemented with 10% fetal bovine serum, 100 ng/ml streptomycin and 100 U/ml penicillin. All of the cell lines were cultured in a humidified chamber with 5% CO_2_ and at 37°C.

### Quantitative reverse transcription-PCR

Total RNA was extracted using the TRIzol Reagent® (DSBIO, Guangzhou, China) according to the manufacturer's instructions. Complementary DNA (cDNA) was synthesized from 2 µg of total RNA using M-MLV reverse transcriptase (Promega, Madison, WI). cDNA was amplified by AmpliTaq® Gold DNA polymerase (Applied Biosystems, Foster, CA) and gene specific primers. The following primers were used for Cry1, 5′- GAGTATGATTCTGAGCCCTTTG-3′ (forward), 5′-GGTTGTCCACCATTGAGT-3′ (reverse). GAPDH was used as an internal control.

### Western Blot analysis

Total cellular proteins were extracted and separated in SDS-PAGE gels, and Western Blot analysis was performed according to standard procedures. GAPDH was used as a loading control on the same membrane. The primary antibodies that were used included monoclonal anti-Cry1 (1∶1000, Abcam, USA) and anti-GAPDH (1∶2000, Santa Cruz Biotechnology, USA). Proteins were visualized using the ECL procedure (Amersham Biosciences, USA).

### Cell transfection

The coding sequence of Cry1 was amplified and cloned into the *NotI* and *BamHI* site of pcDNA3.1^+^ to generate a pcDNA3.1^+^-Cry1 expressing vector; the resulting construct was confirmed by sequencing. Cry1 siRNA (GCAAGAGAAUUUGCUUAAUTT) and control siRNA (UUCUCCGAACGUGUCACGUTT) were purchased from Shanghai Genepharma Co. Ltd. (Shanghai, China).

For transient transfection, SW480 cells (6×10^5^ cells per well) and HCT116 cells (5×10^5^ cells per well) were seeded in 6-well plates at 60% confluence and transfected with 100 nM of oligonucleotides or 4 µg of plasmid, using Lipofectamine 2000 ((Invitrogen) according to the manufacturer's instructions. After 6 h of incubation at 37°C, the transfection medium was replaced with 3 ml of complete medium containing 10% FBS. Cells were collected for western blot, proliferation and invasion assays at different times.

### Retrovirus packing and transduction

Cry1 and controlsequences were cloned into the *Xho*I and *Cla*I site of the pLNCX2 Retrovirus vector. Virus packing was performed in GP293 cells. GP293 cells were cultured in DMEM with 10% FBS in a 37°C incubator with 5% CO_2_. Forty-eight hours after transfection, the supernatant was collected by centrifugation at 1000 g for 10 min. The HCT116 cells were transduced with the retrovirus containing Cry1 or control sequences plasmids. Forty-eight hours after infection, G418(600 ug/ml) was added to the media for 2 weeks to select the stable cells infected with the retrovirus. Western blotting assays were used to detect the expression of Cry1 in two stable cell lines as described above.

### Cell viability assay

The 3-(4, 5-dimethylthiazole-2-yl)-2, 5-biphenyl tetrazolium bromide (MTT) assay was used to detect cell proliferation. HCT116 cells were plated in 96-well at 1×10^3^ cells/well. The spectrophotometric absorbance of each sample was measured at 490 nm. All experiments were repeated three times, and the average results were calculated.

For the colony formation assay, HCT116 cells (4×10^3^ cells per 10 cm^2^ plate) overexpressing Cry1or control GFP and SW480 cells (5×10^3^ cells per 10 cm^2^ plate) down-regulated for the expression of Cry1 by siRNA or a negative control were seeded in complete medium. The cells were cultured for 14 days at 37°C in 5% CO_2_ humidified air. Colony formation and growth were visualized by crystal violet staining. The numbers of colonies containing >50 cells were determined, and 12 fields were counted.

### *In vitro* migration assay

The migration ability of cells was measured in transwell chambers (8 µm pore; BD Biosciences, Franklin Lakes, NJ). The bottom chamber was filled with 700 µl of DMEM containing 10% FBS. For the migration assay, tumor cells (1×10^5^ cells in a total volume of 200 µl) were placed in the upper chamber and incubated at 37°C in 5% CO_2_ humidified air. After 24 h cultured, non-migrating cells on the upper surface of the membrane were removed, and the cells that migrated to the underside of the polycarbonate membrane were fixed with ethanol and stained with crystal violet for 10 min. The number of migrating cells was then determined from 5 independent microscopic fields. The mean of triplicate assays for each experimental condition was used for analysis.

### Immunohistochemical assay

The expression of Cry1 in primary tumors and adjacent noncancerous colorectal mucosa were examined using an immunohistochemical assay. The immunohistochemical assay was performed within five days of section preparation. Paraffin sections were cut to a thickness of 4 um and mounted on silanized slides. The sections were then dewaxed, rehydrated and blocked with 0.3% hydrogen peroxide. Tissue antigens were retrieved with a microwave oven set at 95°C for 25 minutes and cooled to room temperature in 10 mmol/sodium citrate buffer (pH 6.0). Each slice was then washed with phosphate-buffered saline (PBS) and incubated overnight at 4°C with Cry1 (1∶400, clone ab54649, abcam, Cambridge, UK). Primary antibodies were diluted by background reducing components (S2022, Dako, Glostrup, Denmark). The secondary antibody was employed with the Envision Detection Kit (Dako). The slides were stained for 2 min with diaminobenzidine tetrahydrochloride (DAB) and then counterstained with hematoxylin. Tissue treated with antibody dilution solution was used as a negative control. All controls yielded satisfactory results.

### Evaluation of staining

The specimens were evaluated by two investigators, who were unaware of the clinical outcome and who independently reviewed the stained slides. Each slide was assessed under microscopy at ×200 magnification. The expression of Cry1 was evaluated using H-scores. The H-scores consisted of an assessment of the intensity of staining and the percentage of the staining area having a given intensity. Only stained malignant cells were assessed. The samples were grouped into the following four categories based on the intensity of nuclear staining: none (0), weak (1), medium (2) and strong (3). The indexed sum was obtained by multiplying the intensity grade by the percentage of staining area.

The Cry1 expression level was dichotomized according to DFS and OS by a ROC curve. The cut-off value of the positive rate was the maximized sum of the sensitivity and specificity points.

### *In vivo* proliferation assays

Female athymic BABL/c nude mice (4–5 weeks old) were purchased from the Medical Animal Center Guangdong Province (Guangdong, China). All animal studies were conducted inaccordance with NIH animal useguidelines and the current Chinese regulations and standardson the use of laboratory animals. To determine the proliferation capacity of the cell linesstably high expressing Cry1 *in vivo*, a total of 1×10^6^ cells were injected subcutaneously into the left of nude mice and the negative control group were injected into the right (n = 9). The tumor volume was evaluated using the following formula: tumorvolume = 4π/3×(width/2)^2^×(length/2).Four weeks after injection, the animals were sacrificed.

### Statistical analysis

All data are presented as mean ± SEM unless stated otherwise. A paired *t* test was used to test for the differences in Cry1 expression between matched tumor and benign mucosa. The statistical significance of the *in vitro* studies was analyzed using Student's *t*-test. Kaplan-Meier and log-rank tests of the equality of the survivors were used to draw the survival curves by high versus low Cry1 IHC scores (as defined by the ROC curve). All *p* values were two-sided, and *P*<0.05 was the level for statistically significant. All statistical analyses were conducted using SPSS software package, version 16.0(SPSS Inc., Chicago, IL, USA).

## Results

### Characteristics of patients and tumors

**Figure 1:**
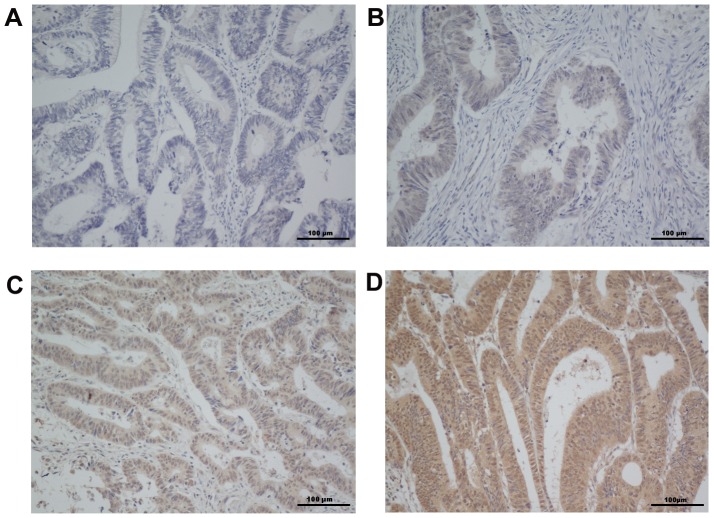
The expression of Cry1 protein in colorectal cancer sections. Representative immunohistochemical images of colorectal cancer tissue specimens indicating negative or weakly detectable Cry1 staining (**A** and **B**); moderate Cry1 staining (**C**); and strong Cry1 staining (**D**) are shown. Magnification is ×200 (**A, B, C** and **D**).

A total of 168 patients who received radical resection between January 1999 and December 2005 were studied. The characteristics of all the patients are summarized in Table 1. The mean patient age was 54.6 years (SD, 14.3). The median follow-up duration was 84 months (range, 4 to 146). Eighty-five tumors (50.6%) were located in the colon. Ten out of the 168 tumors (6.0%) were T1 or T2. Ninety-four tumors were TNM stage I–II (56.0.0%). The median number of harvested lymph nodes was 19 (range from 0 to 48). Cry1 protein expression levels were used for immunohistochemical analysis. Cry1 was stained mainly in the cytoplasm and nuclear regions of the cells. High Cry1 protein expression was detected in 101 samples (60.1%) and weak or negative staining was observed in 67 tumor samples (39.9%, Figure 1).

**Table 1:** Clinicopathological findings and correlation with Cry1 expression.

Variables	No. (%)	Cry1 low	Cry1 high	P value
**Total cases**	168	67(39.9)	101(60.1)	
**Age (years)**				
**≤65**	127(75.6)	47(37.0)	80(63.0)	
**>65**	41(24.4)	20(48.8)	21(51.2)	0.181
**Gender**				
**Male**	89 (53.0)	39 (43.8)	50 (56.2)	0.268
**Female**	79 (47.0)	28 (35.4)	51 (64.6)	
**Tumor location**				
**Colon**	85 (50.6)	34 (40.0)	51 (60.0)	0.975
**Rectum**	83 (49.4)	33 (39.8)	50 (60.2)	
**Tumor size(cm)** †				
**< = 5**	92 (55.1)	33 (35.9)	59 (64.1)	0.285
**>5**	75 (44.9)	33 (44.0)	42 (56.0)	
**Histology**				
**Adenocarcinoma**	146 (86.9)	57 (39.0)	89 (61.0)	0.567
**Mucinous**	22 (13.1)	10 (45.5)	12 (54.5)	
**Tumor invasive depth**				
**T1–T2**	10 (6.0)	6 (60.0)	4 (40.0)	0.180
**T3–T4**	158 (94.0)	61 (38.6)	97 (61.4)	
**Lymph node status**				
**N0**	98(58.3)	48(49.0)	50(51.0)	* 0.004
**N1(n> = 1)**	70(41.7)	19(27.1)	51(72.9)	
**Preoperative CEA(ng/mL)** ††				
**<5**	98(65.8)	38(38.8)	60(61.2)	0.330
**> = 5**	51(34.2)	24(47.1)	27(52.9)	
**AJCC/TNM stage**				
**I–II**	94(56.0)	47 (50.0)	47 (50.0)	* 0.003
**III–IV**	74 (44.0)	20 (27.0)	54 (73.0)	
**Preoperative CA199(ng/mL)** †††				
**< = 35**	118 (80.3)	51(43.2)	67 (56.8)	0.392
**>35**	29 (19.7)	10 (34.5)	19 (65.5)	

* Statistically significant. Numbers in parentheses indicate the proportion of tumors with a specific clinical or pathological feature in a given Cry1 subtype.

† Analysis for this parameter was available for 167 cases.

†† Analysis for this parameter was available for 149 cases.

††† Analysis for this parameter was available for 147cases.

### Cry1 is overexpressed in colorectal cancer tissues

**Figure 2:**
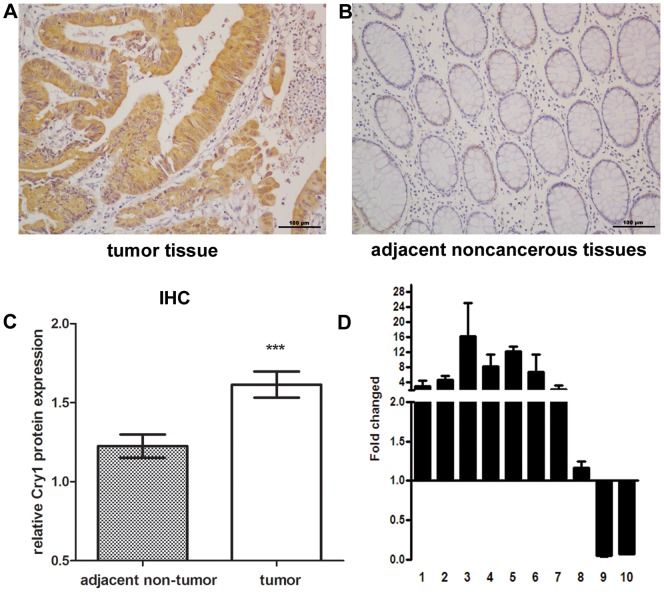
The overexpression of Cry1 mRNA and protein in colorectal cancer tissues. (**A**) A representative image of Cry1 staining in colorectal cancer tissues is shown. (**B**) A representative image of Cry1 staining in adjacent noncancerous tissues is shown. (**C**) Cry1 protein expression level was higher in tumor tissues compared to adjacent control tissue as detected by immunoblotting (mean±SEM; n = 109; * **, *P*<0.001). (**D**) Average T/N ratios of Cry1 mRNA expression in paired colorectal cancer (T) and normalmucosa tissues (N) were quantified by qPCR and normalized to GAPDH. Error bars represent the standard deviation of the mean (SD) calculated from three parallel experiments. Magnification is ×200.

Cry1 mRNA expression in colorectal cancer was investigated using RT-PCR; analyzes of Cry1 mRNA were executed on ten matched pairs of colorectal cancer samples and adjacent noncancerous tissue samples. Cry1 mRNA was expressed at higher levels in eight of the colorectal cancer tissue samples than in adjacent noncancerous tissues. The differential high expression ranged from 1.1-fold to 13.1-fold (Figure 2D). Consistent with these data, Cry1 protein was also up regulated in colorectal cancers compared with the matched control tissue samples (Figure 2A–C).

### Association between Cry1 expression and clinicopathological variables

The association of Cry1 expression and clinicopathological variables was further analyzed. Cry1 expression and all of the other clinicopathological parameters were dichotomized into two groups. The results are summarized in Table 1. There was a significant correlation between Cry1 expression and both the TNM stage (*p* = 0.003) and lymph node status (*p* = 0.004).

Among the 168 colorectal cancer patients, no significant correlation was found between Cry1 expression and gender, age, location of primary mass, tumor size, tumor differentiation grade, histological type, Preoperative CA199 or CEA level (*p*>0.05).

### Association between Cry1 expression and survival

**Figure 3:**
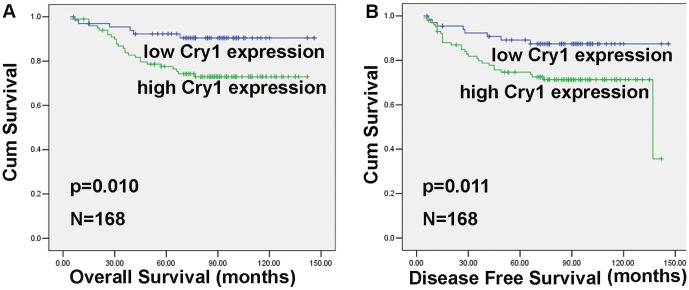
The level of Cry1 protein expression affects overall survival and disease-free survival. Kaplan–Meier curves with univariate analysis (log-rank) for colorectal cancer patients with high Cry1 expression (n = 101) versus low or no Cry1 expression for overall survival (n = 67) (**A**) and disease-free survival (n = 67) (**B**) are shown. Higher expression of Cry1 positively correlated with the poor patientoutcomes.

The application of Cry1 expression as a prognostic marker for CRC patients was also investigated. At the end of the follow-up period (March 1, 2012), 32 patients had died from colorectal cancer-related diseases. The five-year survival rate of DFS was 74.7% for the Cry1 high-expression group. This rate was significantly lower than the survival rate (89.1%) for the low-expression group (*p* = 0.011). Similarly, the five-year survival rate of OS was 77.6% in the Cry1 high-expression group and 92.3% in the low-expression group (*p* = 0.010) (Table 2 and Figure 3).

**Table 2:** Univariate analysis of clinicopathological parameters and molecular expression.

Variables	Disease free survival			Overall survival		
	n‡	5-y(%)	P value	n‡	5-y(%)	P value
**Age (years)**						
**≤65**	26/127	80.6		22/127	81.7	
**>65**	11/41	80.0	0.326	10/41	74.1	0.290
**Gender**						
**Male**	18/89	82.6	0.522	15/89	82.2	0.512
**Female**	19/79	78.0		17/79	77.0	
**Tumor location**						
**Colon**	15/85	84.1	0.197	12/85	84.8	0.112
**Rectum**	22/83	76.6		20/83	74.7	
**Tumor size(cm)** †						
**< = 5**	20/92	77.4	0.790	19/92	77.7	0.446
**>5**	16/75	82.2		12/75	83.5	
**Histology**						
**Adenocarcinoma**	35/146	78.8	0.114	31/146	78.2	0.065
**Mucinous**	2/22	90.9		1/22	95.5	
**Tumor invasive depth**						
**T1–T2**	2/10	90.0	0.786	2/10	78.8	0.927
**T3–T4**	35/158	79.8		30/158	80.7	
**Lymph node status**						
**N0**	14/98	89.4		13/98	85.9	*0.022
**N1(n> = 1)**	23/70	67.9	*0.001	19/70	71.4	
**Preoperative CEA(ng/mL)** ††						
**<5**	13/98	86.0	*0.004	13/98	85.5	0.058
**> = 5**	18/51	70.3		13/51	78.1	
**AJCC/TNM stage**						
**I–II**	10/94	93.4	*<0.001	9/94	89.7	*<0.001
**III–IV**	27/74	63.9		23/74	67.2	
**Preoperative CA199(ng/mL)** †††						
**< = 35**	20/118	86.0	*0.030	17/118	84.1	*0.033
**>35**	11/29	65.5		9/29	69.0	
**Cry1 expression**						
**Low**	8/67	89.1	*0.011	6/67	92.3	*0.010
**High**	29/101	74.7		26/101	77.6	

* Statistically significant.

† Analysis for this parameter was available for 167 cases.

†† Analysis for this parameter was available for 149 cases.

††† Analysis for this parameter was available for 147cases.

‡ The number of positive events/total events. 5-y (%), five-year rate.

### Cry1 is highly expressed in most of the CRC cell lines

**Figure 4:**
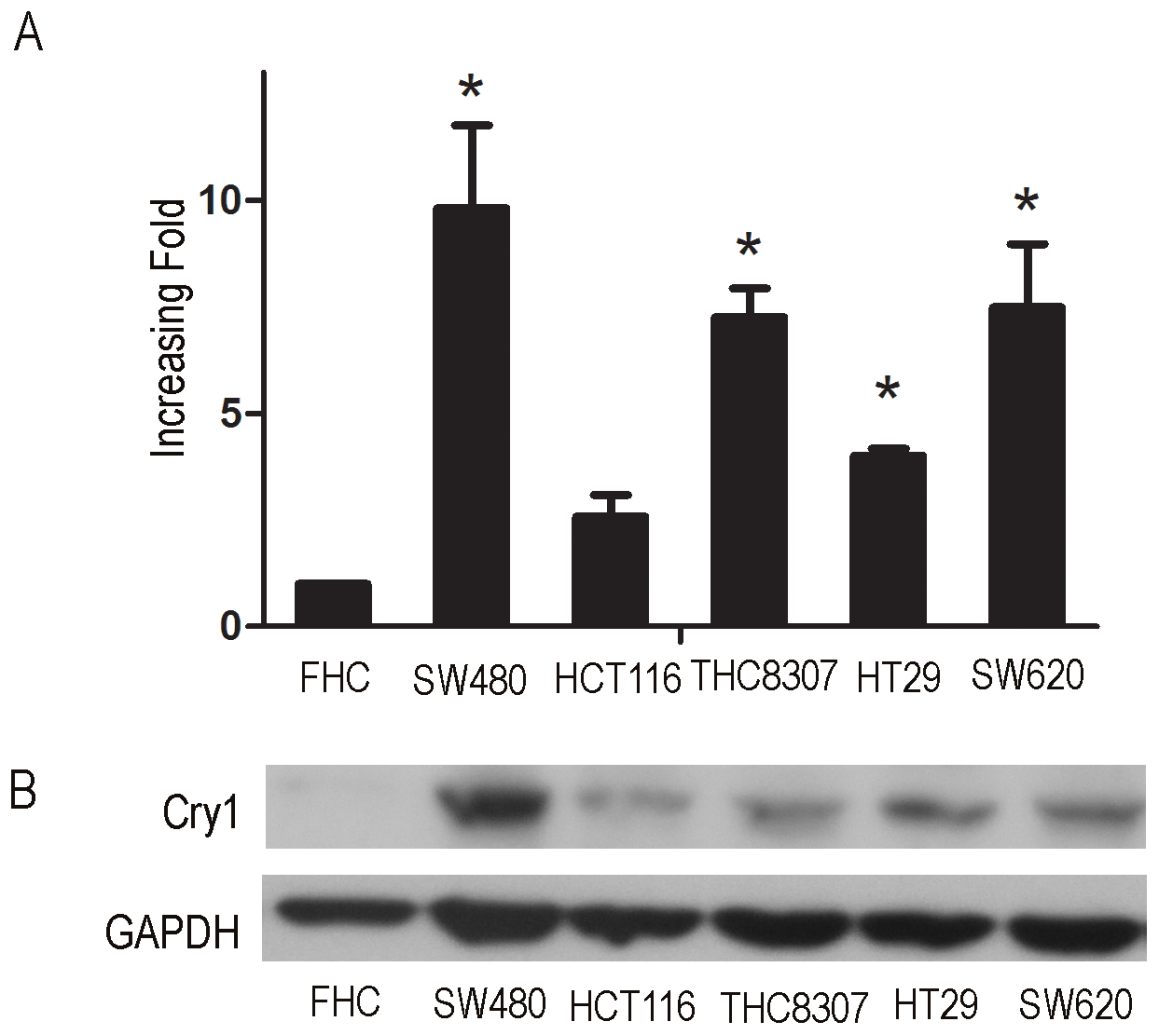
Overexpression of Cry1 mRNA and protein in colorectal cancer cell lines. Expression of Cry1 mRNA and protein in colorectal cancer cell lines (SW480, SW620, HT29, THC8307, and HCT116) and FHC were examined by qPCR (**A**) and Western blotting (**B**). Expression levels were normalized to GAPDH. Error bars represent the standard deviation of the mean (SD) calculated from three parallel experiments, *, *P*<0.05.

To investigate the potential role of Cry1 in the tumorigenesis of colorectal cancer, the expression of Cry1 mRNA and protein was determined for five CRC cell lines (SW480, HT29, SW620, THC8307 and HCT116) and a normal colon epithelium cell line, FHC. Cry1 mRNA expression was at most 10-fold higher in the colorectal cancer cell lines than in the FHC cells (Figure 4A). Cry1 protein was highly expressed in the colorectal cancer cell lines and only weakly expressed in the FHC cells (Figure 4B).

### Cry1 promotes tumor growth and proliferation

Next, we investigated the effects of Cry1 manipulation (through gain-of-function and loss-of-function) on the proliferation of cancer cells.

**Figure 5:**
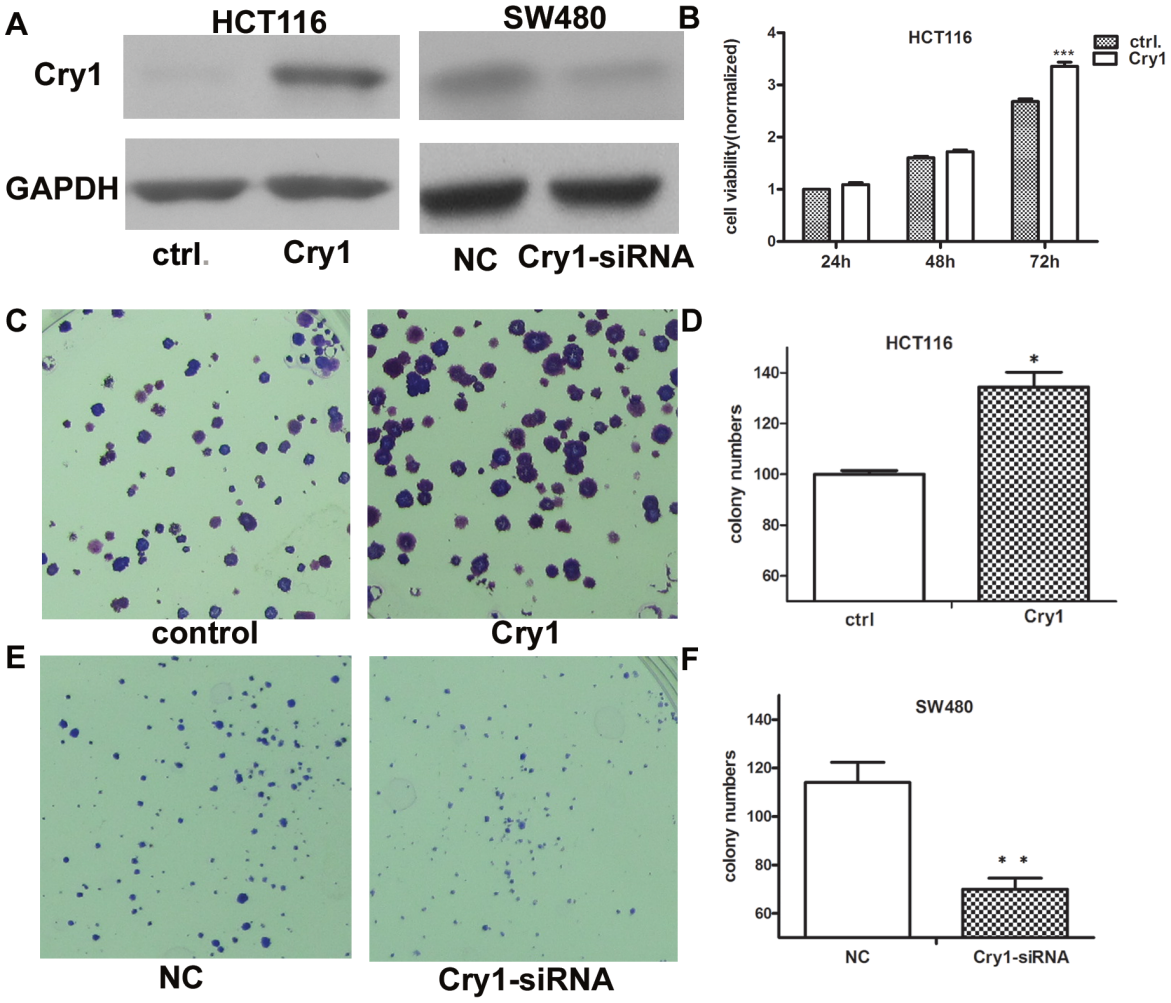
Effects of Cry1 on cell growth. (**A**) Cry1 protein levels were upregulated in HCT116 cells and downregulated in SW480 cells. (**B**) MTT assays on HCT116 cells 24, 48, and 72 h after transfection with Cry1 or GFP control (***, p<0.001). (**C–D**) Colony formation assay of HCT116 cells transfected with Cry1 or GFP control (*, *p* = 0.033). (**E–F**) Inhibition of SW480 cell colony formation capacity by Cry1 siRNA relative to control (**, *p* = 0.007). Experiments were repeated at least three times, and representative data are presented; *bars*, SD.*, *P*<0.05; **, *P*<0.01, ***, *p*<0.001.

We exogenously overexpressed Cry1 in the HCT116 cells, which have a lower endogenous expression. The impact of Cry1 on cellular proliferation was then evaluated using MTT and clonogenic assays. The results showed that overexpression of Cry1 can promote cellular proliferation in instantaneous transfection HCT116 cells (*p*<0.001, Fig. 5B). In contrast, the control group showed no significant effect on cellular proliferation. The colony formation assays also showed that overexpression of Cry1 significantly increased the number of colonies formed after 14 days of culture compared with the control cells (*p* = 0.033, Fig. 5C, D). The link between Cry1 and colorectal cancer cell growth was further established using colony formation assays after Cry1 knockdown. As shown in Figure 5E and 5F, knockdown of endogenous Cry1 expression by siRNA resulted in a dramatic inhibition of clone formation by SW480 cells(*p* = 0.007)

### Cry1 promotes themigration of colorectal cancer cells

**Figure 6:**
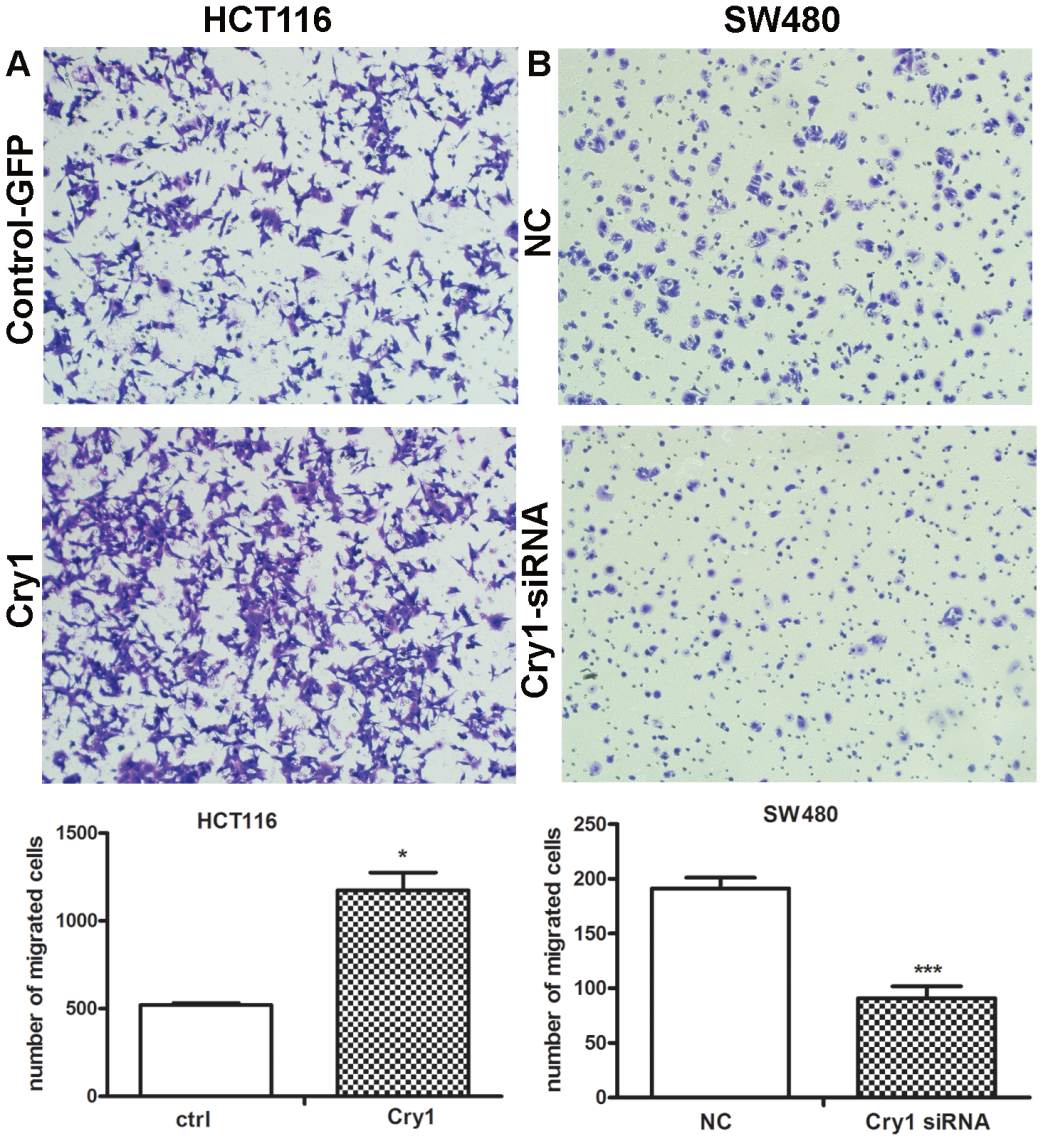
Cry1 promotes CRC cells migration. (**A**) Migration assays of HCT116 cells transfected with Cry1 or GFP control. (n = 3,*, *p* = 0.019) (**B**) Migration assays of SW480 cells transfected with Cry1-siRNA or negative control. (n = 3, ***, *p*<0.001). Representative images are shown above, and the average number of cells per field at the indicated time points are shown below. Data are the mean of three independent experiments.

As for the Cry1 expression was associated with lymph node status, the effect of Cry1 on the migration of colorectal cancer cells was explored using the transwell assay to examine the effects of Cry1 overexpression or deletion. In the transwell assays, HCT116 cells transfected with Cry1 or GFP control plasmids were seeded into the chambers, and their migration potential was determined 24 h after transfection. The assays showed that the migration capacity of HCT116 cells overexpressing Cry1 was increased by 112% compared with the control cells (*p* = 0.019, Fig. 6A).

Confirmation that Cry1 promoted the migration of colorectal cancer cells was assessed by the transfection of SW480 cells with Cry1-siRNA or negative siRNA. After 24 h of culture, the migration capacity of the knock-down Cry1 SW480 cells was reduced by 52.4% compared with the control cells ( *p*<0.001, Fig. 6B).

### Cry1 promoted CRC growth *in vivo*

**Figure 7:**
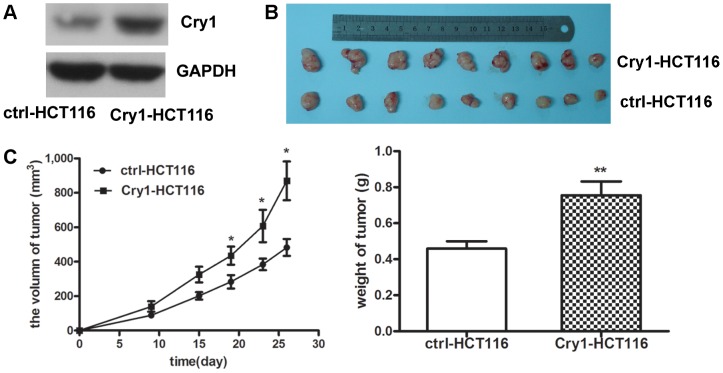
Cry1 promoted CRC growth *in vivo*. (**A**) The expression of Cry1 was markedly increased in the stable cell line Cry1-HCT116 compared with control stable cell line ctrl-HCT116. GAPDH was used as an internal control. (**B**) Representative images of tumors derived from Cry1-HCT116 or ctrl-HCT116,following subcutaneous xenograft transplants in nude mice (**C**) Overexpression of Cry1 promoted colorectal cancer growth. Tumor cells were injected subcutaneously into nude mice. Mice were sacrificed after 4 weeks, and the volume of each tumor was measured every 4 days. Bars, ±SEM; *P<0.05, ** *p* = 0.01.

To further investigate the oncogenic properties of Cry1 *in vivo*, we constructed a retrovirus vector overexpressingCry1 and established two stable cell lines, which were named ctrl-HCT116 and Cry1-HCT116 (Fig. 7A). These two cell lines were injectedsubcutaneously into the flanks of nude mice. Tumor progression was studied over time. At 4weeks post-implantation, the mice were sacrificed, and the tumors were removed. As shownin Fig. 7B–C, the volume and weight of the tumors resulting from injection of Cry1-HCT116 cells were significantly larger and heavier than those originated from the ctrl-HCT116 cells. Takentogether, these observations are consistent with the *in vitro* results and indicate that Cry1 has the ability to promote CRC cell growth *in vivo*.

## Discussion

To our knowledge, this is the first study to evaluate the protein expression levels of Cry1 in human colorectal cancer. Clearly, Cry1 expression was higher in cancerous tissues and cells than that in normal tissues and cells. We also studied the relationship of the expression levels of Cry1 to patient outcomes and clinicopathological features. Our results suggest that overexpression of Cry1 gene could be a useful predictor of lymph node metastasis, TNM stage and poor outcomes in patients with colorectal cancer.

Several previous studies have compared the expression levels of Cry1 between cancer tissue and adjacent normal mucosa. One study found that Cry1 and other circadian gene (Cry2, Per2 and BamlI) mRNA expression levels were similar in colon cancer and adjacent normal mucosa [20]. Another study found that mRNA expression levels of Cry2 and Per2 were down regulated in colorectal cancer. This study also found higher Cry1 expression in the tumor mucosa of cancers located in distal colorectal segments. Cry1mRNA levels in CRC tissues were also significantly associated with patient age and sex [21]. In human epithelial ovarian cancer, Cry1 mRNA expression level was clearly higher in cancerous tissues than in normal tissues [22]. Our findings are in partial disagreement with these studies. In our study, Cry1 gene protein expression levels were higher in cancerous tissues than in adjacent noncancerous tissue. The same results were found in colorectal tumor cells and normal cells, and overexpression of Cry1 was found to promote growth and migration in colorectal tumor cells. However, no significant correlation was found between Cry1 expression and gender, age, location of primary mass, tumor size, tumor differentiation grade, histological type, preoperative CA199 or CEA level (*p*>0.05). These results seem to be reasonable for the following reason. Cry1 protein expression levels may be inconsistently associated with mRNA expression levels for these environmental factors. However, the proteins play the most important role in the biologic effects, and we detect protein expression of Cry1 in CRC tissues.

We also examined the relationships of Cry1 expression with clinicopathological features and patient outcomes. The Cry1 protein levels were significantly associated with the AJCC/TNM stage (with the highest levels detected in TNM stages III–IV) and lymph node involvement (with the highest levels detected in the case of positive lymph nodes). Circadian genes have been implicated in cell cycle regulation [23]. Cry1 mutant mice have an elevated level of Wee1 in many tissues, including the liver. Wee1 kinase blocks cell division by inhibiting the G2-M transition. High Cry1 expression may inhibit the ability of Wee1 to promote cell proliferation, thereby providing a survival advantage for CRC [24].Moreover, the Cry1 protein is known to complex with adenylyl cyclase, and overexpression of Cry1 reduces cAMP production in response to PGE2, isoproterenol, and even the direct adenylyl cyclase activator, forskolin [25]. Studies have reported that high concentrations of cAMP can inhibit the migration and metastasis of human prostate cancer cells. PKA is a protein kinase that is related to the cAMP signaling pathway. Studies have suggested that PKA inhibits the activity and function of RhoA [26], [27]. RhoA is a key member of the Rho family of small GTP-binding proteins and mediates signaling related to cytoskeletal arrangement, migration, proliferation and gene expression [28], [29], [30]. Previous studies have found that invasive growth and metastasis were repressed in a variety of cancer cells (including CRC cells) when RhoA activity was inhibited [26], [31]. The current findings suggest that a Cry1-cAMP/PKA-RhoA mediated pathway is involved in the migration and metastasis of CRC.

Our immunostaining data agree with these studies. Higher TNM stage and the presence of lymph node metastasis were significantly correlated with higher levels of Cry1 expression, suggesting that Cry1 can be used as a marker to determine the progression of CRC. Our future studies will be aimed to more fully dissect the molecular mechanism underlying Cry1 promotion of colorectal tumor cell growth,migration and the progression of CRC.

## Conclusions

In summary, the present study showed that Cry1 expression was up regulated in the majority of the CRC clinical tissue specimens at the protein level. Higher expression of Cry1 positively correlated with the aggressive phenotype of colorectal cancer and predicted poor patientoutcomes. We also present experimental evidence that overexpression of Cry1 in colorectal cancer cell lines promoted cell proliferation and migration. Based on these findings, we conclude that Cry1 is functionally important in the development and progression of colorectal cancer and that Cry1 may serve as a new target for colorectal cancer therapy.
